# *Trypanosoma cruzi*-infected *Rhodnius prolixus* endure increased predation facilitating parasite transmission to mammal hosts

**DOI:** 10.1371/journal.pntd.0009570

**Published:** 2021-07-01

**Authors:** Newmar Pinto Marliére, Marcelo Gustavo Lorenzo, Alessandra Aparecida Guarneri

**Affiliations:** Vector Behavior and Pathogen Interaction Group, Instituto René Rachou, Fundação Oswaldo Cruz-FIOCRUZ, Belo Horizonte, Brazil; University of Iowa, UNITED STATES

## Abstract

Triatomine bugs aggregate with conspecifics inside shelters during daylight hours. At dusk, they leave their refuges searching for hosts on which to blood feed. After finding a host, triatomines face the threat of being killed, because hosts often prey on them. As it is known that many parasites induce the predation of intermediate hosts to promote transmission, and that ingestion of *Trypanosoma cruzi*-infected bugs represents a very effective means for mammal infection, we hypothesized that trypanosomes induce infected bugs to take increased risk, and, as a consequence, be predated when approaching a host. Therefore, we evaluated whether the predation risk and predation rates endured by *Rhodnius prolixus* increase when infected with *T*. *cruzi*. Assays were performed in square glass arenas offering one central refuge to infected and uninfected 5^th^ instar nymphs. A caged mouse was introduced in each arena after a three-day acclimation interval to activate sheltered insects and induce them to approach it. As hypothesized, a significantly higher proportion of infected insects was predated when compared with uninfected ones (36% and 19%, respectively). Indeed, *T*. *cruzi*-infected bugs took higher risk (Approximation Index = 0.642) when compared with healthy ones (Approximation Index = 0.302) and remained outside the shelters when the host was removed from the arena. Our results show that infection by *T*. *cruzi* induces bugs to assume higher risk and endure higher predation rates. We reveal a hitherto unknown trypanosome-vector interaction process that increases infected bug predation, promoting increased rates of robust oral transmission. The significant consequences of the mechanism revealed here make it a fundamental component for the resilient maintenance of sylvatic, peridomestic and domestic cycles.

## Author summary

American trypanosomiasis, an infection affecting more than 7 million people, is caused by the parasite *Trypanosoma cruzi*, a mammal-specific protozoan transmitted to humans by kissing-bugs. The sylvatic cycle of this parasite is exceptional in that both parasite and invertebrate hosts are not specialized, being able to infect a wide range of mammals and feed on an even wider range of vertebrates, respectively. This complex scenario allows parasites to find diverse transmission pathways, as mammal hosts become infected by ingesting infected mammals or bugs, or by mucosal/skin contact with infected bug feces. Most human cases are due to the latter pathway, but human oral infection is relatively frequent and triggers a very aggressive acute phase that can be lethal. The manipulation of intermediate host behavior by parasites is a frequent mechanism promoting host predation and, which in turn induces parasite transmission to final hosts. Even though the oral route is probably the most important means of *T*. *cruzi* transmission in sylvatic environments, the induction of bug predation by behavioral manipulation has not been tested so far. Our study presents clear evidence showing that *T*. *cruzi* promotes higher risk taking in infected bugs, which in its turn induces increased predation rates on them. Interestingly, this impact is not attained by increasing bug foraging activity, but mostly through promoting specifically risky behavior when approaching the host. We suggest that this is a form of behavioral manipulation that evolved because it promotes increased parasite circulation between bugs and mammals.

## Introduction

Triatomines (Hemiptera: Reduviidae) are hematophagous insects that obtain their meals from animals that reciprocally prey on them. To decrease the risk of predation during foraging excursions, these insects take large and sparse blood meals through relatively short interactions, and present most of their activity during the night. Indeed, triatomines stay hidden inside shelters during most of their life, due to accentuated thigmotaxis and negative phototaxis [1]. Several endogenous events, such as the developmental maturation of host-related behavior [2], which includes the regulation of the expression of olfactory receptors [3], and nutritional status [4], trigger coming out of shelters as a response to the detection of host cues [5]. Once outside the shelter, bugs forage to locate a host for most of the dark phase of the daily light cycle. It is worth highlighting that these intervals during which bugs stay close to hosts and attempting to feed are those with increased predation risk.

When feeding on a mammal, triatomines can become infected with *Trypanosoma cruzi*, a protozoan that is the etiological agent of Chagas disease. This severe infection affects 6–7 million people worldwide, the vast majority of whom live in the Americas [6]. The parasite can also be pathogenic to triatomines, but its virulence depends on factors such as environmental temperature, bug nutritional status and parasite life history [7–12].

Diverse pathogens are known to promote increased transmission rates by altering the behavior of intermediate hosts (see revision in [13–16]). Indeed, these alterations of behavior can promote predation by definitive hosts. In the case of *T*. *cruzi*-triatomine-mammal interactions, the oral route, i.e., ingesting parasites by either eating infected bugs or other mammals, is the most effective means for *T*. *cruzi* transmission, probably responsible for most transmission events sustaining sylvatic and peridomestic cycles [17]. Nevertheless, whether trypanosome infection induces behavioral alterations, imposing negative consequences, remains unknown by lack of experimental data. In fact, the only evidence available in this sense indicates that *T*. *cruzi*-infected *Mepraia spinolai* present enhanced foraging parameters, e.g., higher speed of approach to a host [18].

Studies in this context are essential since altered foraging patterns would modify the spatial and temporal distribution of infected insects and their interactions with mammal hosts. In a previous study, we have shown that *T*. *cruzi*-infected nymphs present decreased non-oriented locomotory activity during the first hours of the scotophase [19], an interval during which healthy bugs initiate foraging [5,20]. The present work evaluates whether *T*. *cruzi* infection increases bug predation rates by inducing increased risk taking in infected insects.

## Methods

### Ethics statement

All experiments using live animals were performed in accordance with FIOCRUZ guidelines on animal experimentation and were approved by the Ethics Committee in Animal Experimentation (CEUA/FIOCRUZ) under the approved protocol number LW 61/12. The protocol is from CONCEA/MCT (http://www.cobea.org.br/), which is associated with the American Association for Animal Science (AAAS), the Federation of European Laboratory Animal Science Associations (FELASA), the International Council for Animal Science (ICLAS) and the Association for Assessment and Accreditation of Laboratory Animal Care International (AAALAC).

### Organisms

The *R*. *prolixus* colony used in our study originated from insects collected in Honduras in the 90’s. Insects were maintained by the Vector Behavior and Pathogen Interaction Group at the René Rachou Institute. Experimental bugs were fed citrated rabbit blood obtained from CECAL (Fiocruz, Rio de Janeiro, Brazil) offered through an artificial feeder at 37°C, alternating with blood from anesthetized chickens. Chickens were anesthetized with intraperitoneal injections of a mixture of ketamine (20 mg/kg; Cristália, Brazil) and detomidine (0.3 mg/kg; Syntec, Brazil). The colony was maintained at 26±1°C, 65±10% RH and exposed to a natural illumination cycle.

The *T*. *cruzi* CL strain, originally isolated from naturally infected *Triatoma infestans* [21] was used to infect the triatomines. Parasites were cultured by twice a week passages in LIT (liver-infusion tryptose) medium supplemented with 15% fetal bovine serum, 100 mg/ml streptomycin and 100 units/ml penicillin. The cultured parasites were passed through triatomines and mice every four months to maintain strain infectivity.

### Triatomine infection

Second instar nymphs were fed on an artificial feeder containing heat-inactivated (56°C, 30min) citrated rabbit blood and a suspension of culture epimastigotes of *T*. *cruzi* (1x10^7^ parasites/ml) as described before [10,22]. This amount of parasites was chosen to ensure infection success, as we have previously shown that in the association *R*. *prolixus*- *T*. *cruzi* CL strain, more than 80% of the parasites are killed in the first 24 h after infection [23]. This reduction was also observed in other studies [24,25]. Infection was confirmed during the 4^th^ instar by urine examination under an optical microscope. Nymphs used for the control group were fed heat-inactivated citrated rabbit blood only. Unfed 5^th^ instar nymphs were used for experiments 30 days after ecdysis in order to grant their motivation for foraging [5].

### Experiments

#### Use of shelters

The activity associated with the use of shelters by *R*. *prolixus* was recorded by means of an infrared-sensitive video camera (Panasonic digital video camera-recorder, model AG-DVC30P) according to the methodology described by [20,26], with modifications. The assays were conducted in a square glass arena (40 x 40 x 20 cm) that presented an artificial shelter in the central area. The shelter was made of a piece of corrugated cardboard (20 x 10 cm) folded in half in order to create a 10 cm^2^ refuge with two lateral accesses approximately 0.5 cm high. Two identical experimental arenas were placed side by side to allow the simultaneous evaluation of healthy and infected nymphs. For each arena, 50 nymphs were released and allowed to acclimatize for 72 h. Four replicate assays were conducted for each treatment (N = 200 per treatment). After this interval, all nymphs found outside the shelters were removed and the number of individuals used for the assay recorded. A mouse (weighing ~40 g) held in a cylindrical plastic container (10 cm high x 8 cm in diameter) closed with a perforated plastic cap was placed in each of the arenas. The container prevented physical contact between nymphs and mouse but allowed chemical (odors) and physical (vibration and heat) stimuli to be emitted, signaling the presence of a host. Mice were placed in the arenas three hours before the end of the initial photophase and remained there throughout the scotophase until three hours after the start of the subsequent photophase. Mice received water and food *ad libitum* during this interval. The video camera was located above in a central position to record insect movement in both arenas. The video records were analyzed for the following parameters: a) percentage of nymphs inside shelters after acclimation, b) percentage of nymphs inside shelters right before introducing the host, c) percentage of nymphs outside shelters in the presence of the host, and d) percentage of nymphs that remained outside the shelters three hours after host removal. During the photophase, fluorescent tubes located overhead illuminated the chamber at a light intensity of ca. 160 LUX. Room temperature was kept at 24±1°C and 12:12 L/D.

#### Predation rates

To evaluate whether trypanosome infection affects bug predation by mammals, the design of the previous experiment was modified to allow contact between nymphs and mice. For this, the mouse was kept inside a steel cage (10 x 6 x 10 cm) that allowed the nymphs to feed on it. Nevertheless, the new design also allowed the mouse to eat bugs that approached it. Assays were analyzed for the following parameters: a) nymphs inside the shelter after acclimation; b) percentage of nymphs inside the shelter right before introducing the host, c) percentage of predated nymphs, d) percentage of nymphs that remained outside the shelters three hours after host removal, and e) percentage of nymphs that succeeded in feeding. Fed nymphs were transferred to a BOD chamber (26±1°C) to evaluate molting rates. We also calculated the number of nymphs that remained close to the host by counting the number of individuals which were less than 1.5 cm from the cage during the last five minutes of each hour; these values were normalized by the number of nymphs in the beginning of the assay. Five replicate assays were conducted for each treatment (N = 250 per treatment).

#### Statistical analysis

A GLM model (repeated measures ANOVA) was used to determine whether infection status affected the percentage of insects found outside shelters during host presentation. Data were arcsine square root transformed before the analysis. The approximation indexes obtained for both treatments were compared by means of a Mann-Whitney test. The total number of insects found inside/outside shelters, as well as the number of predated insects seen in both treatments were compared by means of a Chi-square test. The level of significance was set to α≤0.05.

## Results

### Use of shelters

**Fig 1:**
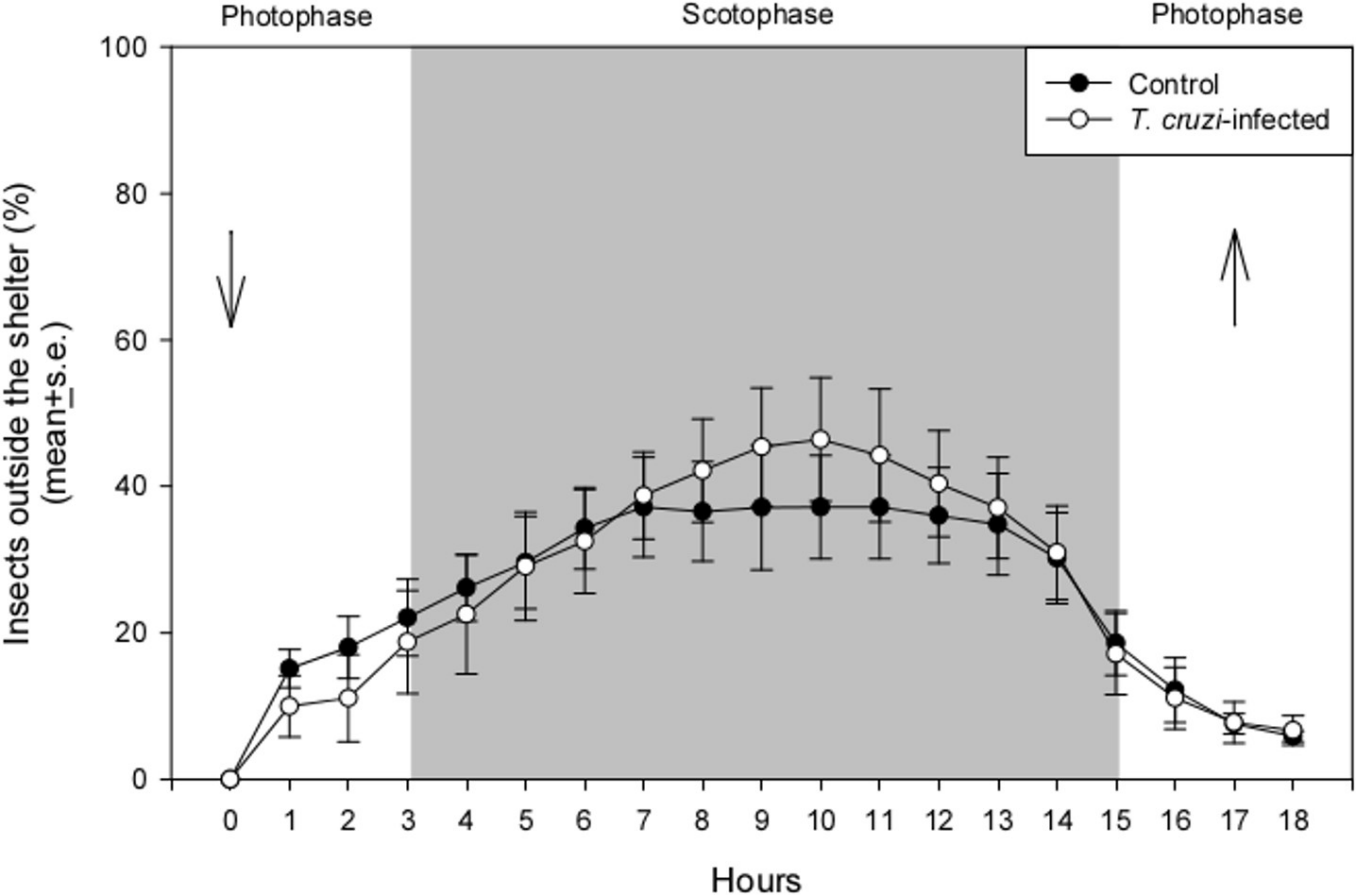
Percentage of *Trypanosoma cruzi*-infected and uninfected *Rhodnius prolixus* nymphs found outside shelters over time. Arrows represent host introduction and removal. Data depicted represent the mean ± s.e. of four independent assays.

Nymph activity in the arena during the three-day acclimation period was similar for both groups (S1 Fig). An intense movement of individuals entering and exiting the shelters was observed during the first six hours after their release. Afterwards, locomotory activity was reduced, with a small proportion of nymphs moving in and out of the shelters. At the end of this period, a similar percentage of insects was found inside the shelters for both treatments (Chi-square, n.s.; 87.5 ± 0.9% for uninfected and 92 ± 0.8% for *T*. *cruzi*-infected nymphs). After placing the host in the arena, the number of nymphs found outside the shelter varied with time, increasing during the scotophase (Fig 1; GLM, p<0.0001). Activity profiles were not altered by *T*. *cruzi* infection (Fig 1; GLM, p = 0.98) and no significant interaction between time and infection was found (GLM, p = 0.44). Around six percent of the nymphs of both treatments remained outside the shelters after host removal (Chi-square, n.s.).

### Predation

**Fig 2:**
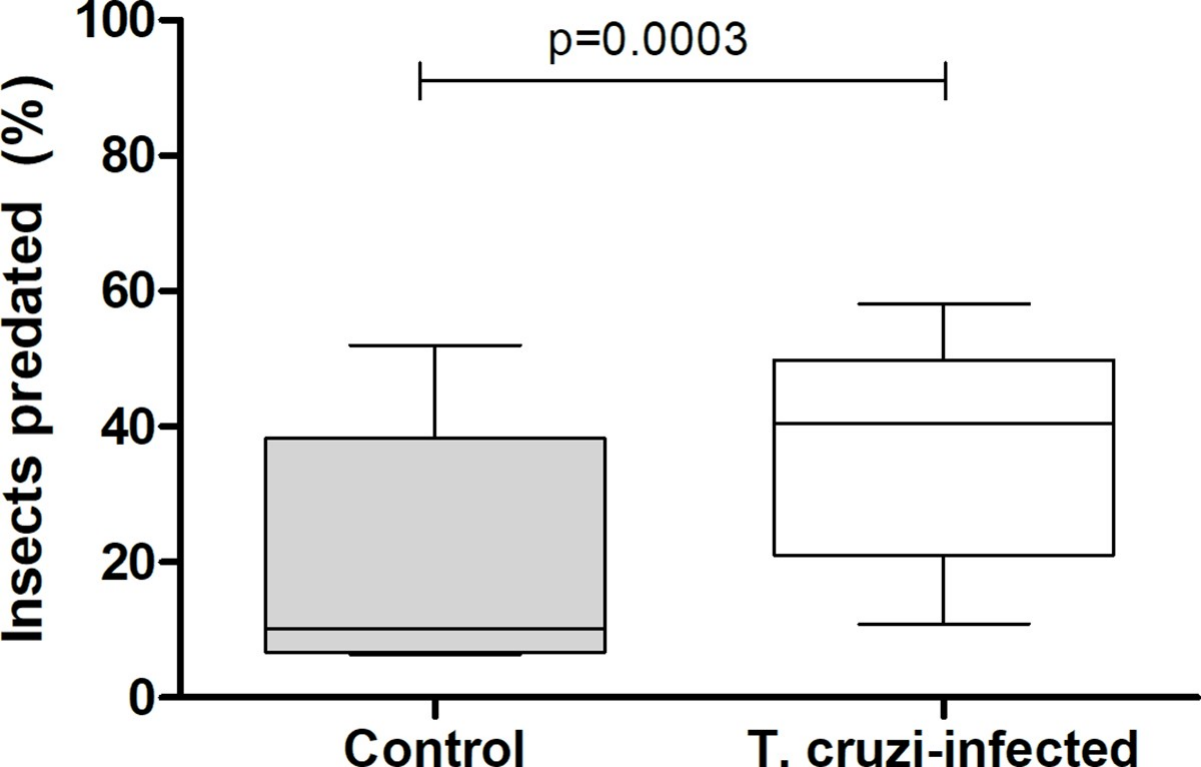
Percentage of *Trypanosoma cruzi*-infected and uninfected *Rhodnius prolixus* nymphs which were predated during the 16 h of exposure to a mouse. Data depicted represent the median (horizontal line) of the percentage of predated insects from five independent assays (25% - 75%, Max—Min).

**Fig 3:**
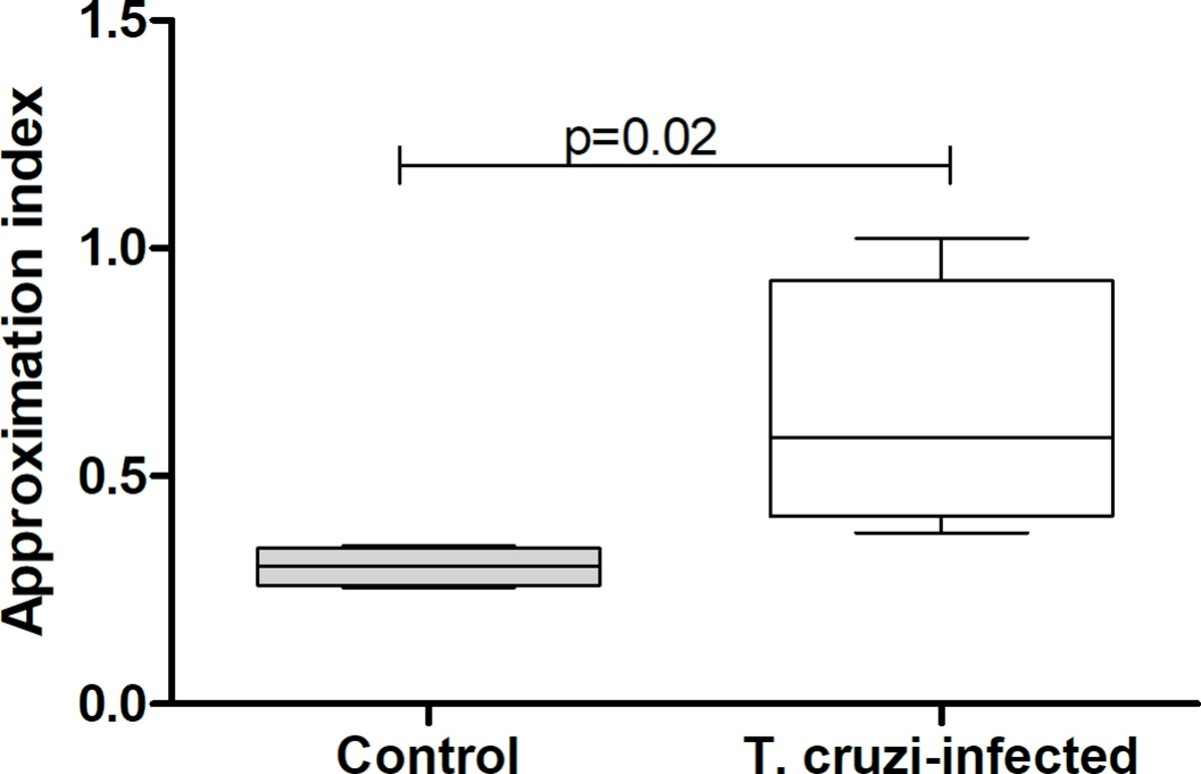
Nymph approximation index comparing the spatial relation between *Rhodnius prolixus* nymphs and hosts, as a function of infection status. Data depicted represent the median (horizontal line) of the cumulative number of nymphs found closer than 1.5 cm from the host in the last five minutes of each recorded hour normalized by the total number of nymphs present in each of four independent assays (25% - 75%, Max—Min).

**Fig 4:**
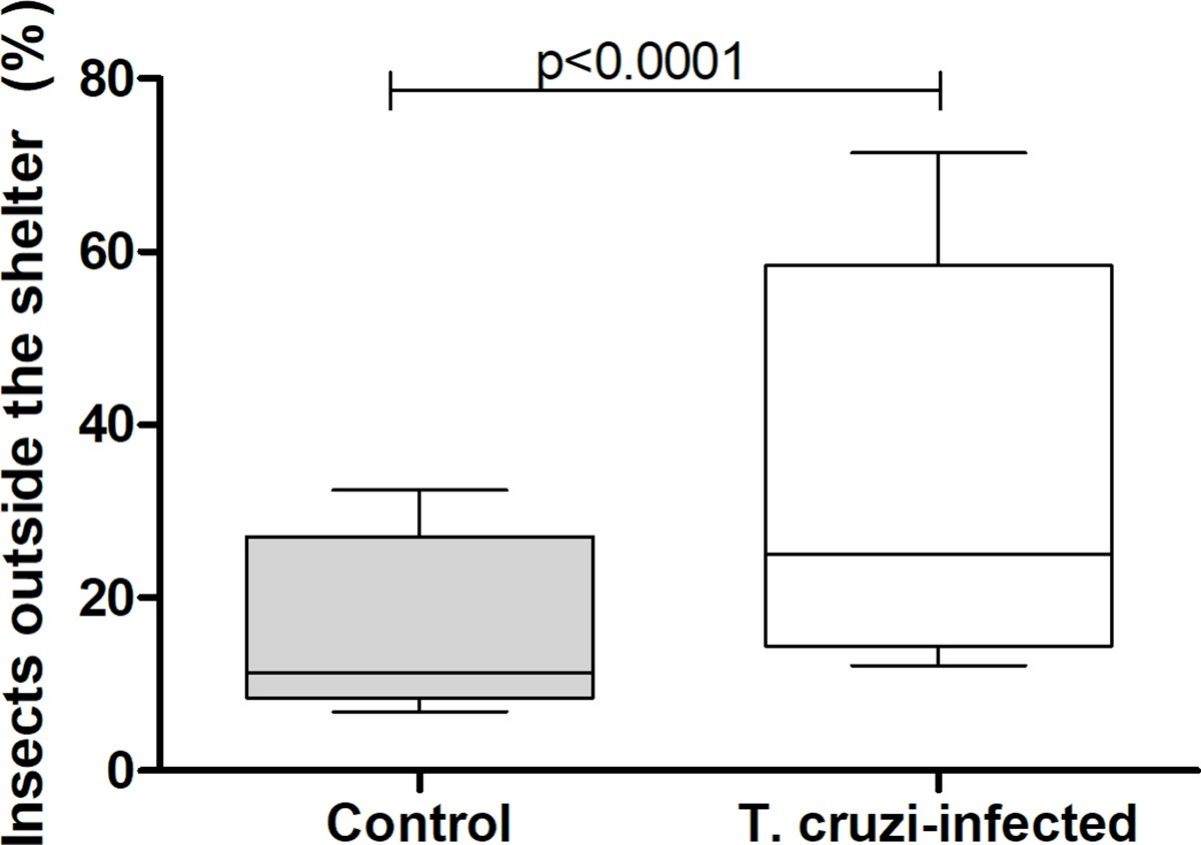
Percentage of *Trypanosoma cruzi*-infected and uninfected *Rhodnius prolixus* nymphs that remained outside the shelter three hours after the host had been removed from the arena. Data depicted represent the median (horizontal line) of the percentage of insects exposed outside shelters in five independent assays (25% - 75%, Max—Min).

Similarl to the previous experiment, 94.4 ± 2.2% and 92.8 ± 1.8% of uninfected and *T*. *cruzi*-infected nymphs, respectively, were found inside the shelters after acclimation (Chi-square, n.s.). Surprisingly, *T*. *cruzi* infection induced a significant increase in the percentage of nymphs predated by the host (20 ± 8.6% for uninfected and 36.4 ± 7.7% for infected nymphs; Fig 2; Chi-square, 14.54, p = 0.0003). The probability of being predated was 1.6 times higher for *T*. *cruzi*-infected insects. Indeed, a significantly higher proportion of infected nymphs remained close to the host during its presentation (Fig 3; Mann-Whitney, p = 0.02). Furthermore, a significantly larger percentage of infected nymphs remained outside the shelter after host removal (Fig 4; Chi-square, 15.52, p<0.0001). Uninfected and infected nymphs presented similar feeding (~19% for both treatments; Chi-square, n.s.) and molting (53% and 68%, for uninfected and infected nymphs, respectively; Chi-square, n.s.) performances.

## Discussion

The survival of triatomines depends critically on the use of shelters because these structures allow bugs to avoid predation [27]. Our study presents significant evidence demonstrating that *T*. *cruzi* infection induces altered bug behavior that translates into higher exposure risk and, consequently, higher predation rates. Based on this, we suggest that increased predation would promote higher parasite transmission rates.

*R*. *prolixus* bugs normally avoid taking risk, as a very low proportion of starved nymphs tends to leave shelters and engage in foraging in the absence of host cues [5]. Nevertheless, introducing host cues in the environment induces a significant increase in this proportion of foraging nymphs, i.e., ~25% [5]. In our study, most nymphs entered the shelters after acclimation. Consistently, few of them were seen foraging during the scotophase, even though it is the period during which triatomine bugs leave their shelters to forage. However, the presence of a mouse emitting multimodal sensory cues including heat, odors and vibration induced a dramatic change, as almost half of the nymphs left shelter protection to forage. Taken together, the previous literature and our results suggest that *R*. *prolixus*, once in a protected place, will only take the risk of becoming exposed if a very strong indication of the presence of a suitable host is perceived.

We have previously shown that the non-oriented locomotory activity of *T*. *cruzi*-infected *R*. *prolixus* nymphs is reduced during the foraging peak [19]. This was proposed as an energy saving mechanism triggered by infection, as in a blood source shortage scenario infected bugs are expected to suffer nutritional deficits. Parasites impose additional nutritional requirements on bugs, as supported by the positive correlation between blood consumption and amount of trypomastigotes found in the rectum in *T*. *infestans* [28]. This idea is reinforced by a recent study that showed that *T*. *cruzi*-infected *M*. *spinolai* captured in the field showed a lower nutritional status [29]. The present results enrich our understanding of triatomine foraging, as infection status did not affect the proportion of nymphs that left the shelter in the presence of host cues. This suggests that both healthy and infected bugs react similarly to the presence of a host. Whether this is also true at the sensory level deserves to be analyzed, as it has recently been shown that dengue-infected *Aedes aegypti* females present larger EAG responses to human odor [30].

The increased predation rates recorded induced us to hypothesize that infected bugs take higher risk when approaching a host, assuming a bolder approach pattern. The significantly higher proportion of infected insects shown to remain close to the mouse suggests that it maybe be a consequence of having decreased nutritional reserves. Additionally, this is reinforced by the higher proportion of infected bugs that remained exposed in the arena after host removal. But, what factor does drive an insect to expose more persistently to predation risk? An increase in biting frequency has been reported for *T*. *cruzi-*infected *M*. *spinolai* [18], an enhanced risk which would seem disadvantageous for *T*. *cruzi*-infected triatomines but beneficial for the parasites. Infection by *T*. *cruzi* is an ancient enzootic condition of wild American mammals in which the most probable strategy for parasite dispersion is the oral route mentioned above, either through the predation of infected triatomines or mammals [17]. Numerous studies have clearly demonstrated that the oral transmission of *T*. *cruzi* is extremely effective [31–34] and recent outbreaks of human oral infection reinforce the importance of this pathway [35–37]. It is worth to emphasize that the stercorarian mode of *T*. *cruzi* transmission is quite inefficient, as it is estimated that 900–4000 bug-host contacts are necessary for an infection event to occur [38]. Our results suggest that the increased rates of predation undergone by *T*. *cruzi-*infected bugs might be a consequence of an underlying mechanism that was selected because if favors parasite transmission. If this hypothesis proves correct, this would not only grant increased parasite transmission in sylvatic and peridomestic *T*. *cruzi* transmission cycles but could also increase parasite transmission in the domestic cycle. Dogs are the main domestic reservoirs of *T*. *cruzi* [39], being a preferred host over chickens and cats [40]. A study testing the use of deltamethrin-treated collars on dogs as a means to decrease *T*. *infestans* infestation suggested that dogs predated on 12% of exposed bugs [41]. This predatory behavior was considered epidemiologically relevant because it would increase the infection rates of a main reservoir of domestic cycles [41]. It is important to highlight that a bolder bug behavior did not translate into improved foraging success, as a similar proportion of surviving bugs fed on mice notwithstanding their infection status (and then a similar proportion of bugs molted to the adult stage).

Behavioral alterations are only considered as evidence of manipulation by parasites when the latter are shown to produce molecules that induce host behavior to change [42]. Thus, our results do not yet prove that *T*. *cruzi* manipulates the foraging behavior of *R*. *prolixus*. Therefore, it is still necessary to determine which physiological mechanisms promote stronger risk exposure in bugs. In addition, and considering that our colony originated many years ago, it would be interesting to test whether this behavioral alteration triggered by infection would still be observed in sylvatic specimens. Considering that an increase in infected bug predation will certainly enhance parasite transmission, these findings present a new look at trypanosome/triatomine-mammal interaction dynamics and open a new avenue for the understanding of the resilient properties of American trypanosomiasis cycles.

## Supporting information

S1 Fig Dynamics of shelter use, i.e., incoming and exiting insects, by *Rhodnius prolixus* during the acclimation interval. The figure depicts the results obtained in a single assay evaluating whether *T*. *cruzi* infection influences the parameters depicted. The white and gray areas represent the photophase and scotophase, respectively.
(DOCX)
