# Supplementary figures and images for: Trypanosoma cruzi-infected Rhodnius prolixus endure increased predation facilitating parasite transmission to mammal hosts
Source: PLoS Negl Trop Dis. 2021 Jul 1;15(7):e0009570. doi: 10.1371/journal.pntd.0009570 (PMC8279422; doi:10.1371/journal.pntd.0009570)

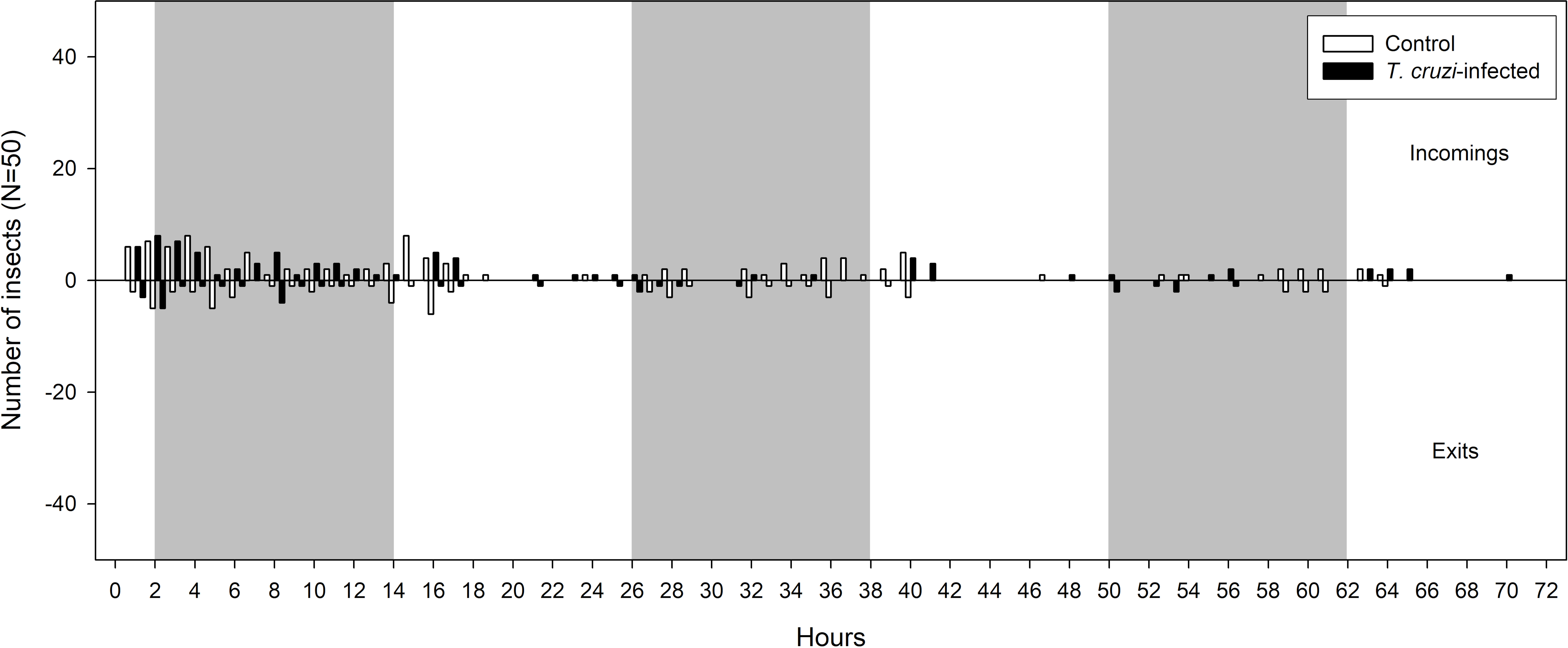

Supplement: S1 Fig — The figure depicts the results obtained in a single assay evaluating whether T. cruzi infection influences the parameters depicted. The white and gray areas represent the photophase and scotophase, respectively. (DOCX) [file pntd.0009570.s001.docx]
